# A Review on the Carbonation of Steel Slag: Properties, Mechanism, and Application

**DOI:** 10.3390/ma17092066

**Published:** 2024-04-28

**Authors:** Shuping Wang, Mingda Wang, Fang Liu, Qiang Song, Yu Deng, Wenhao Ye, Jun Ni, Xinzhong Si, Chong Wang

**Affiliations:** 1 College of Materials Science and Engineering, Chongqing University, Chongqing 400045, China; shuping@cqu.edu.cn (S.W.); wangmd981225@hotmail.com (M.W.); xiaoliu@yeah.net (F.L.); songq231029@hotmail.com (Q.S.); 202109021173t@stu.cqu.edu.cn (Y.D.); ywh0851@hotmail.com (W.Y.); wangchnx@126.com (C.W.); 2 Baowu Environmental Technology Wuhan Metal Resources Co., Ltd., Wuhan 430082, China; e70176@baosteel.com; 3 Shanghai Baosteel Energy Conservation and Environmental Protection Technology Co., Ltd., Shanghai 201999, China

**Keywords:** steel slag, carbonation, soundness, carbonation mechanism, influencing factors

## Abstract

Steel slag is a by-product of the steel industry and usually contains a high amount of f-CaO and f-MgO, which will result in serious soundness problems once used as a binding material and/or aggregates. To relieve this negative effect, carbonation treatment was believed to be one of the available and reliable methods. By carbonation treatment of steel slag, the phases of f-CaO and f-MgO can be effectively transformed into CaCO_3_ and MgCO_3_, respectively. This will not only reduce the expansive risk of steel slag to improve the utilization of steel slag further but also capture and store CO_2_ due to the mineralization process to reduce carbon emissions. In this study, based on the physical and chemical properties of steel slag, the carbonation mechanism, factors affecting the carbonation process, and the application of carbonated steel slag were reviewed. Eventually, the research challenge was also discussed.

## 1. Introduction

Steel slag is a by-product generated during the steelmaking process, with a production rate of approximately 10–15% of the crude steel quantity [1]. Large amounts of steel slag have been produced annually in many regions [2]. As a major steel producer in the world, crude steel production in China reached 1.01 billion tons in 2022 while producing more than 160 million tons of steel slag [3]. However, the comprehensive utilization rate of steel slag in China is still at a low level, which not only occupies a large amount of land but also poses great hazards to the environment due to the dust generated during the transportation and processing of steel slag and the leaching of harmful components in steel slag such as chromium (Cr) and vanadium (V) [4,5].

To recycle steel slag, many studies have been conducted on the properties and potential application of steel slag in different fields, such as stable layer in the subgrade, a supplementary cementitious material or an aggregate in cement and concrete, developing blocks, producing microcrystalline glass, sintering material, soil remediation, and adsorption of heavy metals in sewage, etc. [6,7,8,9,10]. However, the current effective comprehensive utilization rate of steel slag in China is only approximately 30% because of its unsoundness induced by the gradual hydration of f-CaO and f-MgO, and the low hydraulic activity limits its utilization as well [11,12,13,14]. Visible volumetric expansion would occur due to the hydration of these low reactivity phases, i.e., f-CaO and f-MgO, during the service process. Therefore, eliminating the negative effect of f-CaO and f-MgO during the service life of steel slag-based construction materials became the key problem, and pretreatment of steel slag before it is used in the practical application has been considered.

**Figure 1:**
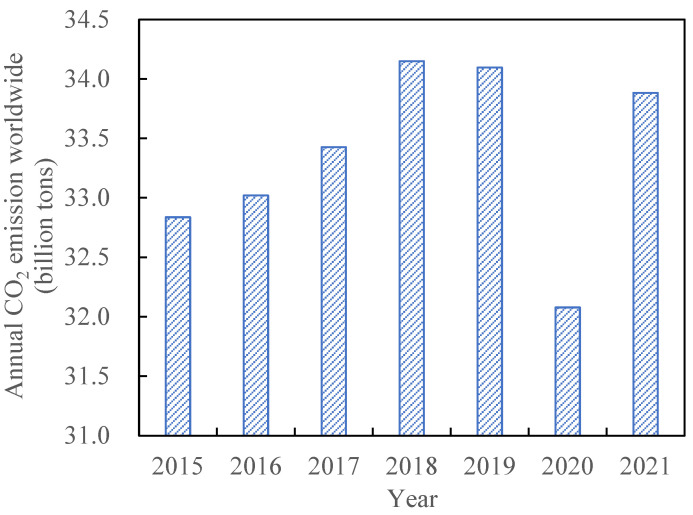
Global CO_2_ emissions from 2015 to 2021 (million tons) [19,20].

As is known, steel slag contains a large amount of silicate minerals, aluminosilicate minerals, and f-CaO and f-MgO. All these phases can react with CO_2_, and the reaction between CO_2_ and f-CaO (or f-MgO) to form CaCO_3_ (or MgCO_3_) during the pretreatment, reducing the instability risk at long-term service in the practical application [15,16]. Furthermore, it can reduce the emission of CO_2_ in the atmosphere and protect the ecological environment, showing a great advantage of CO_2_ capture [17,18]. As reviewed in 2022, the global carbon dioxide (CO_2_) emissions increased by 0.9% or 321 million tons compared with that of 2021 (Figure 1), and the CO_2_ concentration is supposed to be 1000 ppm by 2090 [19,20]. Using carbon compensation technology to reduce the carbon footprint has become a hot topic nowadays, and carbonation treatment is one of the most reliable and economical approaches [21,22,23,24]. It has been reported that the carbon capture, utilizationand storage (CCUS) by steel slag are thermodynamically favored, showing significant prospects [25,26].

Therefore, this article reviews the carbonation of steel slag based on its physicochemical properties, and factors affecting the thermodynamic and kinetic processes of carbonation are discussed. Eventually, the potential application of carbonated steel slag is summarized.

## 2. Physical and Chemical Properties of Steel Slag

According to the production process, steel slag can be divided into converter (BOF) slag, electric arc furnace (EAF) slag, ladle furnace (LF) refining slag, stainless steel (AOD) slag, etc. Among them, BOF slag is one of the main types of steel slag [11,27]. In China, more than 80% of steel slag is derived from the converter process [28].

Due to the high iron content, BOF slag is a dark rock-like substance with an angular surface and a sponge-like interior, showing an aggregate pattern. The specific gravity of BOF slag usually ranges from 3.35 to 3.42 g/cm^3^, and it was reported that the physical properties of steel slag aggregate were superior to those of crushed limestone aggregate [14,29]. The water absorption of steel slag was between 2.0 and 3.31%, the crushing index was approximately 21%, and the soundness index evaluated by the mass loss after immersed in the sodium sulfate solution reached 16% [30]. Steel slag exhibits poor grindability attributed to the presence of ferrite phase. Xiang et al. [31] reviewed that due to the high content of iron oxide and MgO-MnO-FeO solid solution (RO) phase and the high absolute density with a value of approximately 3.7 g/cm^3^ on average, the grindability of converter steel slag was poor.

Steel slag mainly consists of CaO, SiO_2_, Fe_x_O_y_ (Fe_2_O_3_/FeO/Fe), Al_2_O_3_, and MgO, with the remaining minor oxides such as MnO, P_2_O_5_, Na_2_O, SO_3_, etc., which are similar to those of Portland cement [32,33]. However, due to the diversity of iron ore, additives, steelmaking methods, and cooling processes, the chemical composition of BOF slag fluctuates greatly, as shown in Table 1. BOF steel slag can be divided into low-alkalinity, medium-alkalinity, and high-alkalinity steel slag based on the basicity, a weight ratio of CaO to the sum of SiO_2_ and P_2_O_5_. Steel slag with a basicity of 0.78–1.8 is generally considered as low basicity steel slag, a value of 1.8–2.5 is referred to be medium basicity, and a value of 2.5 or higher is referred to as high basicity steel slag. The content of tricalcium silicate and RO phase in steel slag varies depending on basicity. With respect to BOF slag, it usually has a relatively high basicity, with a value of approximately 2.5–5, and the main mineral phases were C_3_S, C_2_S, C_4_AF, C_2_F, and RO [34,35,36,37,38]. Therefore, the ground steel slag powder can be used as a cementitious material, but the hydraulic potential was poor due to the formation of coarse and dense crystals during the slow cooling process of melted slag [39]. In addition, the formation of the RO phase and transformation of β-C_2_S to γ-C_2_S during cooling also reduced the reactivity of steel slag. On the other hand, owing to its high content of belite (C_2_S) and lime (CaO), BOF slag is prone to react with CO_2_ to produce carbonation products at certain temperature ranges [40].

## 3. Carbonation of Steel Slag

### 3.1. Carbonation Mechanism of Steel Slag

**Figure 2:**
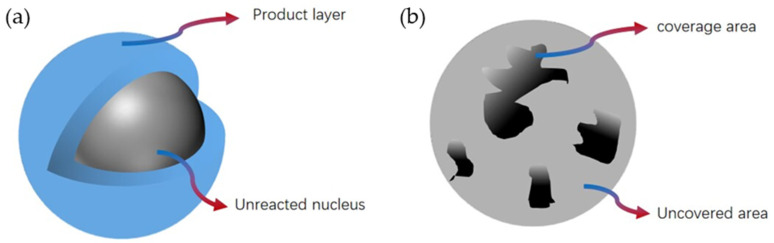
Schematic diagrams of two kinetic models: (**a**) unreacted core model, (**b**) surface coverage model (modified according to [48]).

The carbonation reaction process of steel slag is similar to that of lime carbonation. Research conducted by Wei [48] has shown that the reaction occurs from the surface of the particle initially. Once CO_2_ is in contact with the mineral phase of steel slag, carbonation products form on the particles immediately. As the reaction proceeds, the carbonation layer becomes thicker and denser, preventing the diffusion of CO_2_ into the particles. Finally, unreacted reaction cores may form, as shown in Figure 2a. The carbonation reaction of steel slag includes the following steps: (1) diffusion of CO_2_ gas to the surface of steel slag; (2) diffusion of CO_2_ gas via the CaCO_3_ product layer; (3) CaO reacts with CO_2_ gas at the reaction interface, and the reaction interface moves inward [49].

**Figure 3:**
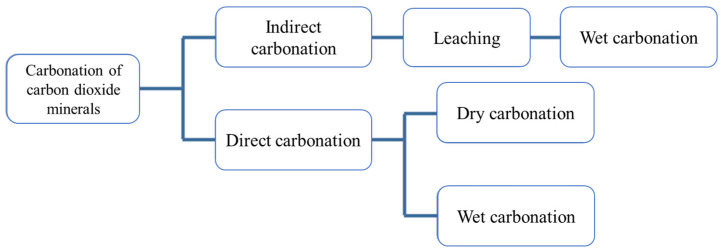
The main carbonation approaches of steel slag [44,53].

**Figure 4:**
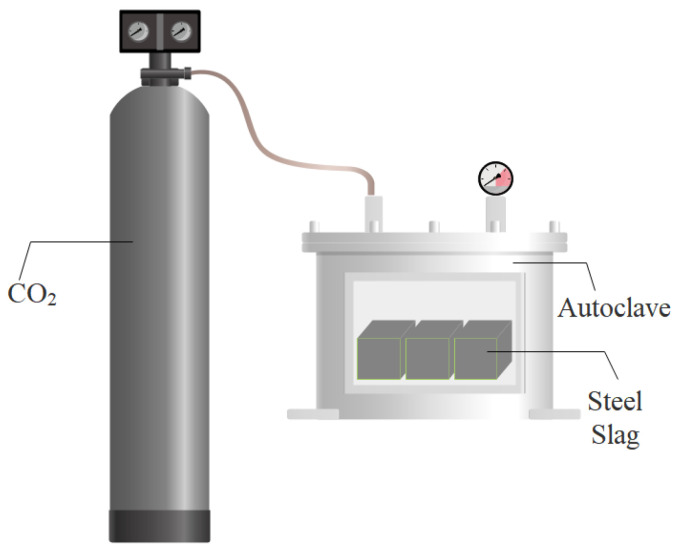
Dry carbonation method of steel slag (according to [54]).

**Figure 5:**
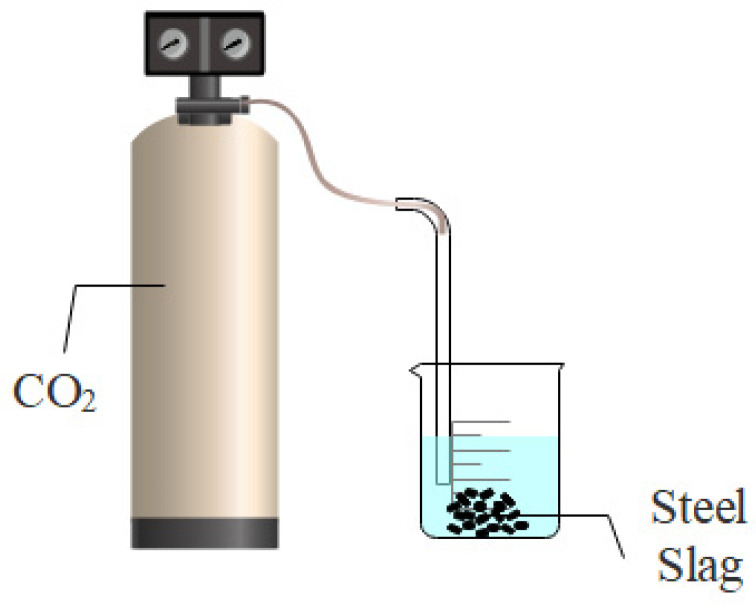
Wet carbonation method of steel slag in aqueous solution (according to [50,51]).

However, because of the uneven distribution of the mineral phase, the carbonation products also distribute heterogeneously on/in the particles. A surface coverage model was proposed to describe the carbonation product distribution on the surface of the steel slag particles, as shown in Figure 2b. It is believed that the reaction only occurs on the unreacted active surface sites, and as the reaction time increases, the reaction continues with the products covering the active surface. Due to the similarity of the reactions, this model is also applicable to the reaction between Ca(OH)_2_ and CO_2_. The calcium-containing phase reacts with CO_2_ to form calcium carbonate, which adheres to the surface of steel slag, resulting in the coverage of the active surface [48]:

The carbonation kinetic process on the steel slag particles is controlled by the reaction between dissolved Ca^2+^ and CO_2_ in the initial stage, and it is then related to the diffusion of CO_2_ molecules in the carbonated layer. In general, the carbonation methods of steel slag are different, including indirect carbonation and direct carbonation, as shown in Figure 3. In terms of indirect carbonation, the carbonation was conducted after the extraction of alkali metals that CO_2_ is immersed into the steel slag slurry. Direct carbonation can be divided into dry carbonation (Figure 4) and wet carbonation (Figure 5), depending on the water content in the steel slag, and the reaction occurs in a single process step [50,51,52].

Indirect carbonation initially extracts certain metal elements from steel slag using extraction solvents and then injects carbon dioxide gas into the aqueous solution to form carbonate precipitates. Taking cement and concrete as an example, acidic media is used to recover Ca^2+^ ions, which are released from the hydrolysis of clinker (e.g., (Ca(OH)_2_) or from the Ca-O-Si network of hydration products. The leached Ca^2+^ caGition can react with the CO_3_^2−^ anion, which is derived from the dissolve of CO_2_ in the liquid to form calcium carbonate. Due to the low solubility of calcium carbonate, it is prone to precipitate, and consequently, carbonate production was developed. The precipitation obtained by this method showed certain economic value, but the process was too complicated, and the additional chemical solvent undoubtedly increased the cost of treatment [55,56].

Direct dry carbonation is similar to natural weathering, where f-CaO, f-MgO, Ca(OH)_2_, Mg(OH)_2_, C_2_S, and C_3_S in steel slag directly react with CO_2_, as shown in the following chemical Formulas (1)–(6) [57,58,59]:

(1) $${CaO}_{\left(s\right)}+{CO}_{2(g)}\to {CaCO}_{3(s)}$$

(2) $${MgO}_{\left(s\right)}+{CO}_{2(g)}\to {MgCO}_{3(s)}$$

(3) $${Ca(OH)}_{2(s)}+{CO}_{2(g)}\to {CaCO}_{3(s)}+H_{2}O_{\left(l\right)}$$

(4) $${Mg(OH)}_{2(s)}+{CO}_{2(g)}\to {MgCO}_{3(s)}+H_{2}O_{\left(l\right)}$$

(5) $${1/3(3CaO•SiO_{2})}_{\left(s\right)}+{CO}_{2(g)}\to {CaCO}_{3(s)}+{1/3SiO}_{2(s)}$$

(6) $$1/2(2{CaO•{SiO}_{2})}_{\left(s\right)}+{CO}_{2(g)}\to {CaCO}_{3(s)}+{1/2SiO}_{2(s)}$$

**Figure 6:**
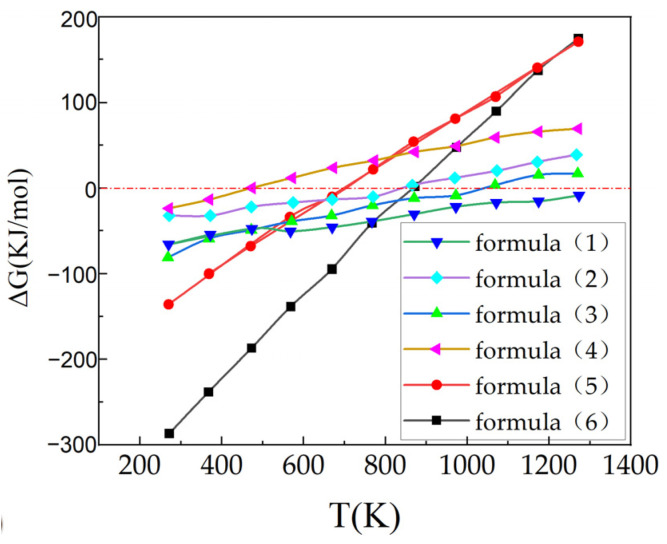
Gibbs free energy (ΔG) of reaction as a function of temperature (according to Tu et al. [57]).

The Gibbs free energies of the above chemical reactions are negative, indicating that the reactions can proceed spontaneously [58]. Tu et al. [57] conducted a thermodynamic simulation on the carbonation process of water-quenched steel slag using the thermodynamic modelling, which also conformed to the above viewpoint. The results present in Figure 6 show that the Gibbs free energy of all the above reaction is negative at the temperature lower than approximately 200 °C. As the temperature increased, decomposition of hydroxide or carbonate products would probably occur.

**Figure 7:**
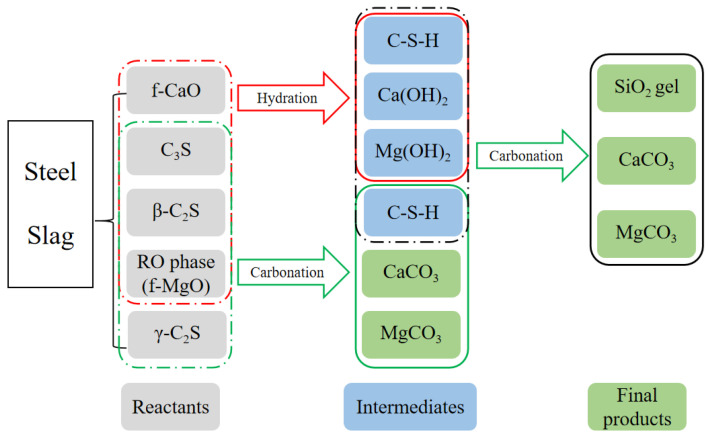
Reaction process of calcium-containing components in steel slag (according to [60]).

Compared with direct dry carbonation, direct wet carbonation is a complex three-phase reaction process, but it can achieve high CO_2_ sequestration at room temperature under atmospheric pressure. This reaction process mainly reflects the carbonation of calcium (magnesium) silicate in solution. The components are involved in the reaction between CO_2_ and hydration products of mineral phases in steel slag, including the hydration of free-CaO, free-MgO, C_3_S, and β-C_2_S. Initially, CaO and MgO hydrate to form Ca(OH)_2_ and Mg(OH)_2_; C_3_S and β-C_2_S hydrate to form C-S-H and Ca(OH)_2_. These phases further react with CO_2_ to produce CaCO_3_, MgCO_3_ and silica-rich C-S-H gel [57,59]. On the other hand, CO_2_ dissolves in water in the form of H_2_CO_3_ to react with C_3_S, and β-C_2_S and γ-C_2_S. Consequently, CaCO_3_ and C-S-H gel forms. With respect to the reaction above, Ca(OH)_2_, Mg(OH)_2,_ and C-S-H are intermediate products, and if there is sufficient CO_2_, decalcification occurs that CO_2_ reacts with the intermediate hydration products to form CaCO_3_, MgCO_3,_ and silica gel eventually. The reaction process of calcium-containing components in steel slag is summarized in Figure 7.

### 3.2. Internal Factors Affecting the Carbonation of Steel Slag

From the above reaction equations (Equations (1)–(6)) and the literature [61,62,63], it can be concluded that the carbonation of steel slag can be roughly summarized as three steps: dissolution of CO_2_ molecules; ionization of Ca, Mg; and precipitation of carbonation product. Therefore, it can be concluded that the internal factors affecting the carbonation process of steel slag are mainly the chemical composition, mineral composition, and particle size of steel slag.

#### 3.2.1. Mineral Phases

A study conducted on the carbon sequestration capacity of the main mineral phases of steel slag and the mechanism of compressive strength growth during carbonation curing showed that Ca(OH)_2_ and γ-C_2_S exhibited the highest capacity to combine CO_2_, whereas the carbonation effects of mayenite, lepidocrocite, and kyanite were relatively poor [38]. However, the compressive strength of the carbonated steel slag specimen was not positively correlated with carbon sequestration. The authors proposed specific strengths of different minerals in steel slag to characterize the carbonation ability of each mineral phase. Results showed that the carbonation of β-C_2_S, when combined per unit weight of CO_2_, contributed most to the strength gain, while the carbonation of Ca(OH)_2_, capturing the same weight of CO_2_, showed the lowest strength value. Other studies showed that under the same carbonation conditions and similar grain size, the higher the content of calcium-containing components, especially CaO and Ca(OH)_2_, the faster the carbon sequestration rate and the better strengthening effect [64,65].

Apart from the carbonation of C_3_S and C_2_S to form CaCO_3_ and C-S-H gel with different C/S ratios, hydration of steel slag during the pretreatment or storage process would also produce C-S-H gel with different Ca/Si ratios and C-S-H with a high Ca/Si ratio had a faster carbonation rate to absorb more CO_2_ [66,67]. During the carbonation process, decalcification of C-S-H occurs, resulting in the formation of Q^3^ and Q^4^ units of Si-O tetrahedral with a higher polymerization degree, accompanied by volume shrinkage and microcracks growth, and consequently the compressive strength of the carbonated specimen decreased, although a large amount of CO_2_ was absorbed during the carbonation process of C-S-H [38,68,69]. It means that excessive carbonation shows a negative effect on the strength development of the specimen. Methods for monitoring the carbonation degree are important in preparing carbonated steel slag specimens.

#### 3.2.2. Particle Size

The particle size is another important factor that affects the carbonation degree of steel slag. For a certain mass of solid particles, a smaller particle size will bring a larger specific surface area, and the contact surface between the solid and liquid or gas phases is larger, resulting in a higher degree of carbonation reaction [70]. Tu et al. [57] studied the carbonation effect of different particle sizes of steel slag under the conditions of CO_2_ flow rate of 600 mL/min, liquid/solid ratio (L/S) of 10, and temperature of 60 °C. The results showed that at the average particle size of 204.4 μm, 85.4 μm, and 17.1 μm, the carbon sequestration amounts of the steel slag were 2.6%, 5%, and 27.9%, respectively. This result demonstrates that steel slag with finer particle size is more favorable to carbonation. Su et al. [65] performed an experiment and showed that the carbonation degree was 16.3%, 27.2%, 46.3%, and 71.1% when the steel slag with particle sizes of 2~3.5 mm, 1~2 mm, 0.5~1 mm, and smaller than 0.5 mm, respectively, was subjected to 100 °C under a CO_2_ pressure of 250 kg/cm^2^ with a water/slag ratio of 5. Huijgen et al. [71] conducted the carbonation of steel slag with a particle size of smaller than 38 μm at the temperature of 200 °C and CO_2_ partial pressure of 20 bar, and found that the carbonation degree of steel slag was up to 75%.

### 3.3. External Factors Affecting the Carbonation of Steel Slag

Apart from the internal factors, the carbonation of steel slag was also affected by the external factors, including carbonation period, carbonation temperature, CO_2_ partial pressure, etc.

#### 3.3.1. Carbonation Period

The carbonation period is a key factor in practical application. Extending the carbonation time can increase the carbonation depth [72], and the carbonation degree increases as well. He [73] studied the effect of carbonation time on the carbonation degree of steel slag with different particle sizes by spraying phenolphthalein solution indicator on the carbonated samples. There is no doubt that the carbonation depth of steel slag at one hour was lower than that of carbonation for 4 h. The particles smaller than 0.06 mm showed no color variation, indicating that these particles were thoroughly carbonated.

**Figure 8:**
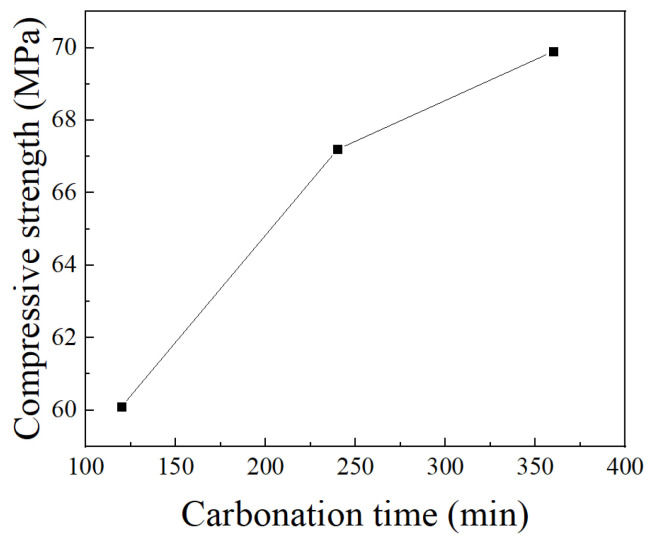
Effect of carbonation time on compressive strength (according to [75]).

**Figure 9:**
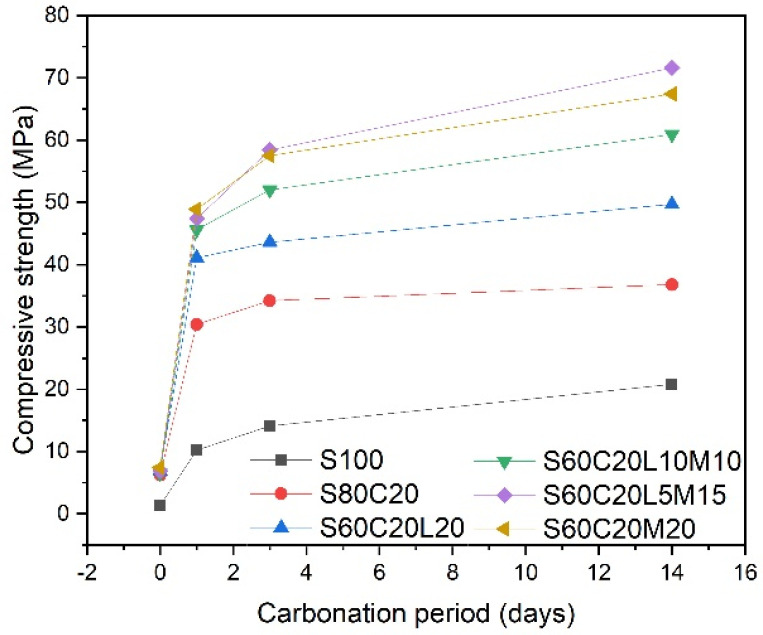
Effect of carbonation period on the compressive strength of steel slag-based mortar (S: steel slag; C: Portland cement; L: CaO; M: MgO) (according to [76]).

Due to the positive correlation between the carbonation rate of steel slag and the compressive strength of steel slag blocks [74,75], characterizing the change in strength with increasing carbonation time can, to some extent, represent the effect of carbonation time on the carbonation of steel slag. In the accelerated carbonation experiment on BOF steel slag blocks conducted by Li et al. [75], results showed that the strength of the blocks increased dramatically by 6.2 MPa as the carbonation period increased from 2 h to 4 h. It was followed by a reduction in strength gain that increased by 2.7 MPa as the carbonation period increased from 4 h to 6 h (Figure 8). As the reaction proceeded, the growth rate of carbon sequestration in steel slag gradually decreased. Moreover, after a long period of time, the influence of carbon dioxide concentration on the carbon sequestration rate of steel slag almost became less pronounced, and even the carbon sequestration rate of steel slag at lower concentrations exceeded that at higher concentrations [75]. Zhang et al. [76] investigated the carbonation of steel slag-based mortar prepared from the mixture of steel slag powder mixed with mineral materials, including MgO, CaO and cement, and found that the compressive strength increased by increasing the carbonation time. The compressive strength of the mortar containing 60% steel slag, 20% Portland cement, 5% lime, and 15% magnesium oxide (S60C20L5M15 in Figure 9) was 47.4 MPa at 1-day carbonation, and it increased to 71.6 MPa when carbonated for 14 days.

In the review above, it can be concluded that prolonging the carbonation period would improve the strength of the specimen, but on the other hand, the contribution to strength development was not so obvious at a long time of carbonation curing. With respect to the carbonation kinetics, the reaction is fast and produces a considerable amount of CaCO_3_ during the initial period of carbonation. As the reaction proceeded, the structure became denser due to the fact of reactants consumption, the filling of pores with CaCO_3_, or the formation of a CaCO_3_ shell on the surface of particles to prevent further penetration of CO_2_ molecules or CO_3_^2−^ ions. Finally, the reaction slows down or even stops.

#### 3.3.2. Carbonation Temperature

The increase in temperature will enhance the thermal mobility of molecules. Generally, increasing temperature can promote the dissolution of active substances in steel slag and the carbonation reaction rate between them and CO_2_ molecules [77,78]. More calcium ions are dissolved from steel slag at higher temperatures, leading to the increasing concentration of calcium ions in the liquid. Consequently, the precipitation of CaCO_3_ at the gas-liquid interface is enhanced. On the other hand, the concentration of dissolved CO_2_ in the liquid phase becomes higher at a relatively lower temperature to promote the formation of CaCO_3_ on the surface of steel slag particles [74].

Luo et al. [79] cured compacted slag steel cylinders in CO2 atmospheres at different temperatures ranging from 0 °C to 90 °C and tested the variation of their compressive strength and CO_2_ absorption ratio. The results showed that during the early stage of carbonization, increasing the curing temperature was beneficial to the development of compressive strength and CO_2_ absorption ratio of the steel slag compacted body. However, as the curing time increased, the strength and CO_2_ absorption of the samples cured at 0 °C and 90 °C were far lower than those cured at 30 °C and 60 °C. According to the kinetic study on the carbonation by Peng et al. [80], the carbonation reaction was accelerated by increasing the temperature, and the highest value appeared at range of 600–700 °C. The carbonation product would be decomposed at a higher temperature. In the same study, it was reported that the carbonation process would be enhanced in the presence of steam pressure.

#### 3.3.3. Partial Pressure of CO_2_

The partial pressure of CO_2_ gas is significant to the carbonation effect of steel slag, as the carbonation reaction of steel slag is related to the dissolution of CO_2_ and calcium ions. According to Henry’s law, the concentration of CO_2_ in the solution is proportional to the partial pressure of CO_2_ above the solution, so when CO_2_ dissolution is the controlling factor to the reaction rate, increasing the partial pressure of CO_2_ can increase the carbonation rate of steel slag. Baciocchi et al. [81] studied the effects of CO_2_ gas concentration and liquid-solid ratio on the carbonation process of steel slag. When 10% CO_2_ gas was introduced, the maximum carbonation amount of steel slag reached 8%, and when the concentration reached 100%, the carbonation amount was 40.3%. Increasing CO_2_ concentration can obviously promote the carbonation degree of steel slag. However, when the dissolution of calcium ions becomes a controlling factor, the influence of CO_2_ partial pressure is not pronounced [60]. The research conducted by Ukwattage et al. [82] supported this view that although the reaction time required for substantial carbonation of steel slag under higher pressure was shorter, no obvious difference in total storage of CO_2_ quantity was observed at the pressures of between 1 MPa and 6 MPa.

In addition, excessively high CO_2_ partial pressure can lead to rapid precipitation of carbonated minerals, blocking pores and forming a shell with a carbonation product on the surface of steel slag particles, preventing further contact between steel slag and CO_2_. Li et al. [75] compared the effects of carbonation temperature, CO_2_ partial pressure, and carbonation time on the mechanical properties and carbonation efficiency of the compacts from compression of EAFS powder and BOFS powder. Results showed that the reaction rate between steel slag and CO_2_ increased as the CO_2_ partial pressure increased to 0.55 MPa, but the compressive strength of the carbonated blocks decreased with further increasing partial pressure. Over-carbonation may have a negative effect on the mechanical properties of the specimen.

Therefore, a suitable partial pressure is required for carbonation curing, but due to the high variability in the properties of steel slag, it is difficult to determine an accurate value for the partial pressure of CO_2_ for carbonation. According to [75], it can be determined that treating a 20 × 20 × 20 mm^3^ steel slag block at a temperature of 70 °C for 30 min under a CO_2_ partial pressure of 0.55 MPa resulted in higher strength of the steel slag block than treating it under a lower partial pressure of CO_2_. It also showed that no further reduction in the strength of the steel slag block occurred with the continuous increase in partial pressure of CO_2_. Furthermore, studies also reported that the carbonation can be increased substantially by increasing the concentration of sodium and potassium bicarbonate solution when the wet carbonation method was applied [83,84].

### 3.4. Soundness of Carbonated Steel Slag

Soundness is quite significant for steel slag and its products, and it is supposed that the soundness of steel slag can be improved by carbonation. The specimen of steel slag carbonated at 0.2 MPa for 7 days exhibited excellent soundness: no obvious defects or microcracks were observed when the specimen was subjected to the autoclave with a saturated steam pressure of 2.0 MPa for 3 h [85]. In the same study, it is shown that the compressive strength reached 70.6 MPa with a porosity reduced to 16.67% [85]. The improvement in the soundness of steel slag by carbonation was because the unsound components, including f-CaO and f-MgO, transformed into CaCO_3_ and MgCO_3_, as demonstrated by Sun et al. [86], who found the reaction between f-CaO/f-MgO and CO_2_ occurred rapidly when the particle sizes of steel slag were smaller than 80 μm. In addition, it is also shown that pretreatment of carbonating steel slag in an autoclave with a CO_2_ concentration of 99.9% at 0.2 MPa for 6 min would greatly reduce the expansive risk of steel slag. Chen et al. [87] reported that when carbonation was adopted, up to 58.83% of magnesium oxide in the RO phase could be converted into magnesium carbonate. The value was much higher than that of MgO transforming into Mg(OH)_2_ under autoclaved curing, which was only 20.10%.

### 3.5. Potential Application of Carbonated Steel Slag

In the review, it has been found that not only the compressive strength of steel slag specimens can be improved by carbonation, but also the risk of unsoundness will be reduced. Therefore, carbonation treatment is an available and reliable method to promote the utilization of steel slag. In general, the carbonated steel slag can be used to prepare bricks, supplementary cementitious materials, and aggregates. The preparation process parameters and performance indicators of certain carbonated steel slag products are summarized in Table 2.

With respect to the utilization of ground steel slag powder, Ye et al. [88,89] prepared carbonated steel slag bricks using steel slag as the precursors and Na_2_CO_3_ solution as an activator , and analyzed the factors that affect the carbonation effect under alkali-activated conditions, including the amount of steel slag, particle size, water consumption, and the type and amount of alkali activator. By comparing the effects of “alkali activation”, “carbonation”, and “alkali activation + carbonation” on the strength, product composition, porosity, and microstructure of the samples, the synergistic effect of alkali activation and carbonation would greatly improve the compressive strength of the bricks. The formation of porous CaCO_3_ to improve the compactness of the brick was the main reason for the increased strength. Hou et al. [90] invented a low-cost method for preparing steel slag bricks on a pilot scale by compressing the steel slag powder and water mixture at 0.16 MPa, followed by carbonation curing under pure CO_2_ with a pressure of 0.2 MPa for different curing periods. The compressive strength of 47.1 MPa was achieved when the bricks were carbonated for 7 days. As an aggregate, steel slag can significantly improve the mechanical properties of concrete, but its utilization is greatly limited due to its presence of expansive components. Several research found that the practical application of carbonated steel slag is possible, once the soundness has been improved. Pang [91] prepared steel slag aggregate by carbonating the granulated steel slag powder and found the crush value of the artificial aggregate was 18.30%. And the concrete prepared from this type of aggregate was approximately 15% to 20%, higher than that produced from natural aggregate.

In addition, carbonated steel slag has also been applied in wastewater treatment [92,93,94], producing high value-added CaCO_3_ [95,96], improving the hydration reactivity as a supplementary cementitious material [97], thermal insulation material [98], soft soil foundation consolidation [99], and artificial stone [100], showing great potential in the realistic application.

**Table 2:** Carbonation parameters of steel slag products and their performance.

Main Materials	Products	Particle Size of Steel Slag	Carbonation System	Compressive Strength (MPa)	Carbon Sequestration Ratio (%)	References
Steel slag powder + sand+ aggregate	Carbonation steel slag brick	—	Carbonation was at the pressure of 0.2 MPa in pure CO_2_ gas for 7 d	27.7	7.5	[90]
Steel slag powder	Carbonation steel slag brick	3–40 μm	the CO_2_ concentration was 98 ± 1%, relative humidity was 60 ± 1%, the temperature was 20 ± 1 °C, the CO_2_ gas pressure was 0.25 MPa, and the carbonation duration was 2 h.	22–32.6	13.28–16.82	[101]
Steel slag powder + pore-forming agent	Steel slag block	<75 μm	Introducing 99.9% mass pure CO_2_ gas to carbonate steel slag at 150 °C for 3 h under the CO_2_ partial pressure of 0.3 MPa	24.8 (1 d)	15.32	[102]
Steel slag powder	Carbonated steel slag cement	Average particle size of 39.4 μm	99.5% purity CO_2_ gas, CO_2_ partial pressure of 1.5 bar for 12 h	39.9–91.2 (12 h)	9–15	[103]
Steel slag powder + Portland cement + reactive magnesia	binding materials	0.036–0.039 mm	CO_2_ with a concentration of 99.9% was introduced for curing at a CO_2_ gas pressure of 0.1 MPa.	38.6 (1 d)	—	[104]
Steel slag powder + sand + cement	Ultra-high performance concrete incorporating carbonated steel slag powder	Steel slag powder < 150 μm/Steel slag fine aggregate	CO_2_ concentration of 20%, temperature 25 °C, curing time 72 h	>145 (28 d)	—	[105]
Steel slag powder + granite	Carbonation steel slag concrete	<50 μm	Introducing tail gas with a CO_2_ concentration of 99.9% at a pressure of 1.4 kPa or 0.5 MPa for 12 h	49.9–54.3 (28 d)	7.3–8.11	[106]
Steel slag powder	Carbonated steel slag aggregate	1–100 μm	Using 99.9% CO_2_, the CO_2_ partial pressure is 0.2 MPa, and the carbonation time is 4, 8 or 24 h	—	7.0–10.5	[107]
Steel slag + biochar	Carbonated steel slag aggregate	—	Curing for 4 h under the reaction temperature of 30 °C and RH of 60 ± 5%, and the pressure of kiln tail gas of 0.2 MPa	3.2–5.7	6.51–8.69	[108]
Steel slag	Carbonated steel slag aggregate	—	Curing with CO_2_ gas with a purity of 99%, RH of 70 ± 5%, curing temperature of 20 ± 2 °C, curing time of 7 h at a gas pressure of 1 bar	20.5	4.49	[109]

## 4. Conclusions and Challenges

### 4.1. Main Conclusion

The carbonation method for treating steel slag not only solves the problem of large-scale application of steel slag but also captures and stores CO_2_ to reduce the emission of greenhouse gas. In this review, different carbonation methods were described. The carbonation mechanism and factors affecting the physicochemical properties of carbonated steel slag were discussed. Finally, the potential application of carbonated steel slag was suggested, and the following conclusions were drawn:

(1) In general, dry carbonation and wet carbonation were commonly used in the treatment of steel slag. Compared with direct dry carbonation, direct wet carbonation is a complex three-phase reaction process and can achieve high CO_2_ sequestration at room temperature. Indirect carbonation would produce pure carbonation products by adding additives, such as hydrochloric acid, sulfuric acid, magnesium chloride, molten salt, acetic acid, and sodium hydroxide, which increases the difficulty of the treatment process.

(2) The carbonation process of steel slag is highly dependent on the chemical composition, mineral composition, and particle size of steel slag. Ca(OH)_2_ and γ-C_2_S absorb the CO_2_ more easily but have different roles in the mechanical properties. Carbonation of β-C_2_S contributes more obviously to the strength development of the specimen, and Ca(OH)_2_ shows less contribution. Reducing the particle size of steel slag can improve its carbonation efficiency. When preparing carbonated steel slag bricks, it is recommended to use steel slag powder with a particle size of less than 38 μm to obtain a high carbonation rate.

(3) Extending the carbonation period can increase the carbonation depth. Increasing temperatures can promote the dissolution of active substances in steel slag and accelerate the carbonation process between the mineral phases of steel slag and CO_2_. However, when the temperature exceeds 700 °C, it will conversely inhibit the dissolution of CO_2_ due to the exothermic reactions during the carbonation process. There is controversy over the effect of CO_2_ partial pressure on the carbonation of steel slag, as the carbonation of steel slag is influenced by both dissolution of CO_2_ and calcium ions.

(4) Carbonation treatment on steel slag can be used to prepare bricks, supplementary cementitious materials, and aggregates in cement and concrete. It can also be applied in wastewater treatment, soft soil foundation curing, fertilizer preparation, artificial stone preparation, GRC board manufacturing, etc.

### 4.2. Future Challenges

However, most of the present research is about the carbonation treatment of steel slag in the laboratory. For the practical application, the following investigation is suggested:

(1) The variability of steel slag components can seriously affect the results of experiments, leading to differences in product performance. Investigation into the relationship between carbonation regimes and the composition of steel slag is necessary.

(2) The carbonation of steel slag is affected by many factors, and there is controversy over the impact of carbonation on the properties of the product, making this treatment uncontrollable. It is necessary to further investigate the carbonation kinetics and thermodynamics, based on modeling and experiments, to establish the relationship between carbonation efficiency and factors, achieving an acceptable treatment cost with relatively high carbonation efficiency.

(3) Apart from consuming f-CaO and f-MgO, the carbonation process also consumes C_3_S, C_2_S, and other minerals with hydraulic potential, thereby reducing the hydration activity of steel slag. Therefore, in order to improve the utilization rate of steel slag, the balance between the soundness and hydration activity of steel slag still needs to be explored.

## Figures and Tables

**Table 1:** Main chemical composition of converter steel slag and cement in China.

Category	Origin	Chemical Composition (wt.%)					Reference
		CaO	SiO_2_	Fe_x_O_y_	Al_2_O_3_	MgO	
Cement	—	64.88	22.08	3.42	4.51	2.28	[41]
BOF1	Beijing	44.21	12	29.74	4.05	4.51	[41]
BOF2	—	40.20	10.76	16.47	4.49	9.48	[42]
BOF3	Panzhihua	42.18	15.02	22.58	6.14	8.94	[43]
BOF4	—	45.34	11.41	30.31	1.31	2.19	[44]
BOF5	—	34.77	26.44	18.40	10.03	6.01	[45]
BOF6	—	40.20	10.76	16.47	4.49	9.48	[46]
BOF7	—	38.48	15.42	26.79	4.45	8.08	[47]

## Data Availability

The data presented in this study are within this article.
